# Molecular and Phenotypic Characterization of *Staphylococcus epidermidis* Isolates from Healthy Conjunctiva and a Comparative Analysis with Isolates from Ocular Infection

**DOI:** 10.1371/journal.pone.0135964

**Published:** 2015-08-14

**Authors:** Luis A. Flores-Páez, Juan C. Zenteno, María D. Alcántar-Curiel, Carlos F. Vargas-Mendoza, Sandra Rodríguez-Martínez, Mario E. Cancino-Diaz, Janet Jan-Roblero, Juan C. Cancino-Diaz

**Affiliations:** 1 Department of Microbiology, Escuela Nacional de Ciencias Biológicas-Instituto Politécnico Nacional, Mexico City, Mexico; 2 Instituto de Oftalmología Fundación Conde de Valenciana, Mexico City, Mexico; 3 Faculty of Medicine, Universidad Nacional Autónoma de México, Mexico City, Mexico; 4 Department of Zoology, Escuela Nacional de Ciencias Biológicas-Instituto Politécnico Nacional, Mexico City, Mexico; 5 Department of Immunology, Escuela Nacional de Ciencias Biológicas-Instituto Politécnico Nacional, Mexico City, Mexico; University of Malaya, MALAYSIA

## Abstract

*Staphylococcus epidermidis* is a common commensal of healthy conjunctiva and it can cause endophthalmitis, however its presence in conjunctivitis, keratitis and blepharitis is unknown. Molecular genotyping of *S*. *epidermidis* from healthy conjunctiva could provide information about the origin of the strains that infect the eye. In this paper two collections of *S*. *epidermidis* were used: one from ocular infection (n = 62), and another from healthy conjunctiva (n = 45). All isolates were genotyped by pulsed field gel electrophoresis (PFGE), multilocus sequence typing (MLST), staphylococcal cassette chromosome *mec* (SCC*mec*), detection of the genes *icaA*, *icaD*, IS256 and polymorphism type of *agr* locus. The phenotypic data included biofilm production and antibiotic resistance. The results displayed 61 PFGE types from 107 isolates and they were highly discriminatory. MLST analysis generated a total of 25 STs, of which 11 STs were distributed among the ocular infection isolates and lineage ST2 was the most frequent (48.4%), while 14 STs were present in the healthy conjunctiva isolates and lineage ST5 was the most abundant (24.4%). By means of a principal coordinates analysis (PCoA) and a discriminant analysis (DA) it was found that ocular infection isolates had as discriminant markers *agr* III or *agr* II, SCC*mec* V or SCC*mec* I, *mecA* gene, resistance to tobramycin, positive biofilm, and IS256^+^. In contrast to the healthy conjunctiva isolates, the discriminating markers were *agr* I, and resistance to chloramphenicol, ciprofloxacin, gatifloxacin and oxacillin. The discriminant biomarkers of ocular infection were examined in healthy conjunctiva isolates, and it was found that 3 healthy conjunctiva isolates [two with ST2 and another with ST9] (3/45, 6.66%) had similar genotypic and phenotypic characteristics to ocular infection isolates, therefore a small population from healthy conjunctiva could cause an ocular infection. These data suggest that the healthy conjunctiva isolates do not, in almost all cases, infect the eye due to their large genotypic and phenotypic difference with the ocular infection isolates.

## Introduction

*Staphylococcus epidermidis* is a common inhabitant of the skin and mucous membranes, however in recent decades it has gained interest due to its high frequency of isolation in hospital acquired infections [1]. The main vehicle to cause infection is by medical device, such as catheters, heart valves, contact lenses, etc. [2–3]. Different strategies have been applied in order to find determinant biomarkers for *S*. *epidermidis* isolates from nosocomial. Some determinant biomarkers include biofilm formation, antibiotic resistance, the presence of the *ica* genes (encoding exopolysaccharide for biofilm formation) which partly explain their opportunistic pathogenicity to humans [4]. However the molecular genotyping of *S*. *epidermidis* isolates by pulsed-field gel electrophoresis (PFGE), multilocus sequence typing (MLST), staphylococcal cassette chromosome *mec* (SCC*mec*) type assignment [5] and the whole genome sequencing [6] have exhibited a high diversity; which makes difficult to discriminate between opportunistic pathogenic strains of *Staphylococcus epidermidis* from the commensal strains.

In the eye, *S*. *epidermidis* can cause infection and the most common examples include conjunctivitis, blepharitis, corneal ulcers and endophthalmitis. The main cause of ocular infection is the use of contact lenses (in the case of conjunctivitis and corneal ulcer) [7] or during ocular surgery (endophthalmitis). High incidences of ocular infection by *S*. *epidermidis* have been reported [8], even in some cases superior than those achieved by *S*. *aureus* [9]. These reports are considerable because *S*. *epidermidis* have not a variety of virulence factors such as *S*. *aureus*. Furthermore, the eye is an organ continuously exposed to the environment, and it is also in contact with various microorganisms. Despite this high exposure, the hazard of ocular infection during a person’ life is low. One possible explanation against bacterial infection in the eye is the production of molecules associated to innate immunity; such as lysozyme, lactotransferrin, secretoglobin family 2A [10], antimicrobial peptides [11], cytokine modulators [12] and TLRs [13], which help to remove the bacteria on the ocular surface.

Additionally, *S*. *epidermidis* inhabits the ocular surface specifically into the conjunctival sac [14]. Innate immunity of the conjunctiva recognizes the commensal population of *S*. *epidermidis*, with specific feature to regulate their growth. *S*. *epidermidis* strains infecting the eye must evade the innate immunity of the host. Probably the virulent pathogenic strains have elements, which would be absent in commensal strains. The origin of infective strains of *S*. *epidermidis* in the eye could be the conjunctiva sac, as in some studies have suggested [15–16], or could be from different origin. The presence of *S*. *epidermidis* in endophthalmitis had shown that the primary source of infection is the healthy conjunctiva [17]. However, in a study of keratitis and endophthalmitis caused by *S*. *epidermidis* was proven otherwise [18]. It has also been documented that *S*. *epidermidis* from healthy conjunctiva is polyclonal because genotyping by PFGE of *S*. *epidermidis* isolates from the conjunctiva of a healthy subject are different [19]. Currently it is unclear the involvement of *S*. *epidermidis* from healthy conjunctiva in ocular infection and the limited research on molecular genotyping of these isolates fail to clarify this point. In this paper, we propose that healthy conjunctiva isolates of *S*. *epidermidis* could provide more information on the source of infective strains to the eye. Therefore, a genetic and phenotypic comparison of *S*. *epidermidis* isolates from healthy conjunctiva and ocular infection may answer this question.

## Materials and Methods

### 2.1 Isolates

*S*. *epidermidis* isolates from ocular infection were obtained from 1995 to 2000 in the Institute of Ophthalmology Conde de Valenciana at Mexico City, DF. Samples were obtained from patients diagnosed with conjunctivitis (n = 21), corneal ulcers (n = 7), endophthalmitis (n = 14) or staphylococcal blepharitis (n = 20). Corneal ulcer samples were obtained by scraping, and conjunctivitis and staphylococcal blepharitis samples were obtained by swabbing. The vitreous samples of patients with endophthalmitis were obtained mainly by vitrectomy. Swabbing samples were obtained from the conjunctiva of healthy individual (n = 45). All clinical samples were spreading onto chocolate blood, and mannitol salt agar plates, and incubated to 37°C for 12 h. For ocular infection isolates, it was considered plates that exhibited single colony morphology and these were re-growth onto mannitol salt agar plates. *S*. *epidermidis* identification was performed by the Vitek Jr computerized system (BioMériux, Craponne, France), using the GPS-101 and V-1305 identification cards for Gram-positive bacteria. Based on the Declaration of Helsinki, all patients signed medical informed consent forms to participate in this study, and the study was approved by the ethics committee of National School of Biological Sciences of the “Instituto Politécnico Nacional” Mexico.

### 2.2 Pulsed-Field Gel Electrophoresis (PFGE)

The *Sma*I DNA restriction fragments were separated by PFGE [20] and the resulting patterns were analyzed using the BioNumerics software (version 4.61 of Applied Maths, Saint-Martens-Latem, Belgium) with previously optimized settings for *S*. *epidermidis* [21].

### 2.3 Genomic DNA extraction

Bacterial cells were grown overnight in tryptic soy broth (TSB; Becton Dickinson, NJ, USA), harvested by centrifugation and resuspended in 200 μL of lysis solution (20% sucrose, 10 mM Tris-HCl pH 8 and 10 μg/mL lysozyme). Cells were incubated at 37°C for 40 min and 200 μL of Whinston solution (2% Triton X-100, 1% SDS, 10 nM NaCl, 10 mM Tris base pH 8.0 and 1 mM EDTA) was added. DNA was extracted with phenol/chloroform/isoamyl alcohol (25:24:1). DNA was subsequently precipitated with a volume of isopropanol and purified by the addition of two volumes of ethanol 70%. Finally, DNA was resuspended in sterile distilled water.

### 2.4 SCC*mec* typing

SCC*mec* typing was performed using the PCR schemes previously published [22–23]. A single PCR was performed for each gene. For isolates in which SCC*mec* could not be typed, classes of the *mec* gene complex and the *ccr* gene complex (*ccr*AB1, *ccr*AB2, *ccr*AB3 and *ccr*C1) were examined by additional PCRs using the primers described previously [22]. SCC*mec* types were assigned based on the *mec* complex classes and the *ccr* gene types according to the criteria set for *S*. *aureus* [22–23].

### 2.5 Molecular Typing by Multilocus Sequence Typing (MLST)

Genomic DNAs of the isolates were subjected to MLST according to the procedure described by Thomas *et al*., (2007) [24]. Briefly, PCRs were performed to amplify fragments (approximately 450 bp) of *arcC*, *aroE*, *gtr*, *mutS*, *pyr*, *tpi* and *yqiL* genes. The amplified products were purified and sequenced by the BigDye terminator fluorescence kit (Applied Biosystems, Foster City, CA, USA). The sequences and allelic profiles assignments were performed through *S*. *epidermidis* MLST database (http://www.mlst.net).

### 2.6 PCR amplification of the *icaA* and *icaD*, and IS256

Genomic DNAs isolates were used as templates for the amplification of *icaA* and *icaD* genes, and IS256 with primers described previously [25]. The PCR reactions were performed with 1 μL of DNA (100 ng), 1X buffer, 1 mM MgCl_2_, 200 μM of each dNTPs, 1 U of Taq DNA polymerase (Invitrogen, Carlsbad, CA, USA) and 0.2 μM of each specific primer. The optimal PCR conditions were 30 cycles of 30 s at 92°C, 40 s at 60°C and 30 s at 72°C. The PCR products were analyzed on agarose gels.

### 2.7 Determination of biofilm formation

A semi-quantitative determination of biofilm formation was performed in 96-well tissue culture plates (Nunc, Rochester, NY, USA) based on the method reported by Christensen *et al*. (1985) [26]. Bacteria were grown in individual wells of 96-well plates at 37°C in tryptic soy broth (TSB; Becton Dickinson, NJ, USA) medium. After 24 h of growth, the plates were washed vigorously with 1X phosphate buffed saline (PBS), dried for 30 min at 55°C, and stained with 0.5% (w/v) crystal violet solution. After staining, the plates were washed with 1X PBS. A_492_ nm of the adhered, stained cells was measured using a Multiskan EX Microplate Photometer (Thermo Fisher Scientific, Lenexa, KS, USA). The criterion outlined by Chistensen *et al*. (1985) [26] was used to determine whether isolates were non-adherent and biofilm-negative (A_492_ < 0.12) or strongly biofilm-positive (A_492_ > 0.12). Assays were repeated six times, and the mean biofilm absorbance values were used. The results were analyzed using a one-way ANOVA with a Tukey’s test.

### 2.8 Genetic polymorphism of *agr* by PCR

Genetic polymorphisms of *agr* were determined according to Li *et al*. (2004) [27]. Briefly, A primers were designed from conserved sequences of *agr*, which are common to *agr* groups I, II and III (1,022 bp). B primers were designed from the hypervarible region, which is common in *agr* groups II and III but not group I (453 bp). C primers are specific for group II (615 bp). The PCR assay was performed following conditions as previously described by Li *et al*. (2004) [27].

### 2.9 Statistical analysis

To analyze the above data, it was used a principal coordinates analysis (PCoA) and then a discriminant analysis (DA), using the PAST program v2.15 [28]. The PCoA is a sorting method, also known as Metric Multidimensional Scaling; PCoA routine calculated eigenvalues (eigenvalues) and eigenvectors (eigenvectors) of a matrix containing Jaccard distances of all data. The routine was processed with default values and the eigenvalues, which provides a measurement of the variance displayed by the corresponding eigenvectors [28]. The discriminant analysis (DA) was carried out, also with default values and the result was used to confirm or reject the hypothesis that the two groups are different. Subsequently, the discriminant function was calculated, which is a linear equation, for classifying data according to the score obtained in the discriminant function. Additionally, the values of the coefficients of the discriminant function can be the contribution of each variable in the DA because the sign variable load (positive or negative) [28] is known. All data phenotypic and genotypic of two isolation sources are included in the supplementary material (S1 and S2 Tables).

## Results

### 3.1 Diversity of STs in strains of *S*. *epidermidis* isolated from healthy conjunctiva and ocular infection

A total of 107 isolates were distributed in 25 STs. Ocular infection isolates (n = 62) were distributed in 11 STs, and lineage ST2 was the most prevalent (30 isolates, 48.4%), followed by ST9 (15 isolates, 24.2%) and ST23 (9 isolates, 14.5%) (Table 1). ST16, ST10, ST87, ST38, ST71, ST21, ST57 and ST46 types were only represented by a single isolate (this group was designated as "Other STs"; Table 1).

**Table 1:** Relationship of ST with genotypic and phenotypic data from the two sources.

STs (n, %)	PFGE types	SCCmec Type (%)	Biofilm + (%)	agr I (%)	agr II (%)	agr III (%)	icaA (%)	icaD (%)	IS256 (%)
**Ocular infection (n = 62)**									
ST2 (30, 48.4)	16	I, II, IV (26.6)	9 (30)	2 (6.6)	14 (46.6)	14 (46.6)	6 (20)	6 (20)	9 (30)
ST9 (15, 24.2)	7	V (46.6)	7 (46.6)	1 (6.6)	4 (26.6)	10 (66.6)	6 (40)	3 (20)	3 (20)
ST23 (9, 14.5)	5	II, IV, V (33.3)	6 (66.6)	0	2 (22.2)	7 (77.7)	2 (22.2)	2 (22.2)	1 (11)
Others STs^a^ (8,13)	2	I (50)	6 (75)	1 (12.5)	2 (25)	5 (62.5)	1 (12.5)	3 (37.5)	2 (25)
**Healthy conjunctiva (n = 45)**									
ST5 (11, 24.4)	10	III (36.4)	0	6 (54.5)	3 (27.3)	2 (18.2)	0	2 (18.2)	1 (9)
ST10 (9, 20)	8	IV (33.3)	0	4 (44.4)	2 (22.2)	3 (33.3)	0	0	1 (11)
Others STs^b^ (25, 55)	24	II (40)	8 (32)	8 (32)	12 (48)	5 (20)	7 (28)	10 (40)	4 (16)

^a^ Represented by ST16, ST10, ST87, ST38, ST71, ST21, ST57 and ST46, each type with a single isolate.

^b^ Represented by lineage ST238 and ST118 (4 isolates for each one, 8.8%), ST2, ST4, ST9 (3 isolates for each one, 6.6%), ST23 (2 isolates for each one, 4.4%), ST26, ST135, ST494, ST43, ST48 and ST173 (one isolate for each one).

In comparing with ocular infection isolates, 14 STs were found in the healthy conjunctiva isolates (n = 45), lineage ST5 was the most prevalent (11 isolates, 24.4%), followed by lineage ST10 (9 isolates, 20%). Lineages ST238 and ST118 (4 isolates of each one, 8.8%), ST2, ST4, ST9 (3 isolates of each one, 6.6%), ST23 (2 isolates, 4.4%), ST26, ST135, ST494, ST43, ST48 and ST173 (1 isolate of each one) had the lowest frequency and they are named as the group "Others STs' (Table 1).

### 3.2 PFGE typing

*S*. *epidermidis* isolates were widely spread in 61 PFGE types. Healthy conjunctiva isolates had more genetic diversity than the ocular infection isolates, 31 PFGE types was presented in healthy conjunctiva isolates, 19 PFGE types in ocular infection, and only 11 PFGE types were shared between both sources of isolation (Table 1). Relationship between ST with PFGE types exhibited a greater diversity of PFGE patterns compared with ST lineages, this implies that PFGE was more discriminatory than MLST and that PFGE increased the genotypic diversity. For the above reason, in this study the STs lineages of the isolates were used to correlate them with additional genotypic and phenotypic data.

### 3.3 Relationship between types of STs and type of SCC*mec*

With regard to ocular infection isolates, the *mecA* gene was 75.8% and SCC*mec* type II was the most recurrent (25.8%). Isolates associated with lineage ST2 from ocular infection were SCC*mec* type I, II and IV (each 26.6%), whereas isolates with lineage ST9 were SCC*mec* type V (46.6%) and lineage ST23 were SCC*mec* types II, IV, and V (each 33%; Table 1). In relation to healthy conjunctiva isolates, the *mecA* gene was 66.6% and SCC*mec* type II (31.1%) was the most recurrent, whereas lineage ST5 was related to SCC*mec* type III (36.4%) and ST10 with the SCC*mec* type IV (33.3%; Table 1).

### 3.4 Relationship between ST, biofilm formation, the presence of *icaA and icaD* genes and IS256 in *S*. *epidermidis*

Ocular infection isolates produced more biofilm (28/62, 45.16%) than healthy conjunctiva isolates (8/45, 17.7%) (p < 0.05). However, ocular infection isolates grouped in "others STs" are the largest biofilm producers, while ocular infection isolates with lineage ST2 were the minor biofilm producers (Table 1). In addition, the isolates included in "other STs" from healthy conjunctiva were the largest biofilm producers in their respective groups (Table 1).

Regarding *icaA* and *icaD* genes, a predominance of 20 to 40% distributed homogenously between the STs lineages of the two sources of isolation were determined, and no statistical significance was shown. Similar results were found with IS256 (Table 1).

### 3.5 Relation between ST and type of *agr* in strains of *S*. *epidermidis*

Ocular infection isolates with lineage ST2 were predominantly *agr* types II and III. Ocular infection isolates with lineages ST9 and ST23 were mainly *agr* type III. ST5 and ST10 from healthy conjunctiva have a tendency to *agr* types I and II (Table 1).

### 3.6 ST relationship with resistance to antibiotics

In general, it was observed that healthy conjunctiva isolates are more resistant to antibiotics than those from ocular infection. Healthy conjunctiva isolates were more multiresistant (14/45; 31.1%) than ocular infection isolates (3/62; 4.8%). Healthy conjunctiva isolates with lineage ST5 were different to ocular infection isolates with lineage ST2, in terms of resistance to oxacillin (p < 0.0058), ciprofloxacin, levofloxacin and chloramphenicol (p < 0.05). In contrast, ocular infection isolates were only resistant to tobramycin (p < 0.05) (Table 2).

**Table 2:** Antibiotic resistance of healthy conjunctiva and ocular infection isolates.

ST (n, %)	Oxa n (%)	Cip n (%)	Ofl n (%)	Lev n (%)	Mox n (%)	Gat n (%)	Tob n (%)	Chl n (%)	Van n (%)
**Healthy Conjunctiva**									
ST5 (11, 24.4)	6 (54.5)	5 (45.4)	4 (36.4)	2 (18.1)	0 (0)	0 (0)	0 (0)	5 (45.4)	0 (0)
ST10 (9, 20)	2 (22.2)	3 (33.3)	3 (33.3)	1 (11.1)	0 (0)	1 (11.1)	0 (0)	2 (22.2)	1 (11.1)
Others STs (25, 55.5)	11 (44)	3 (12)	3 (12)	3 (12)	1 (4)	2 (8)	3 (12)	8 (32)	0 (0)
**Ocular Infection**									
ST2 (30, 48.4)	3 (10)	0 (0)	6 (20)	0 (0)	0 (0)	0 (0)	11 (36.6)	0 (0)	1 (3.3)
ST9 (15, 24.2)	3 (20)	2 (13.3)	4 (26.6)	0 (0)	0 (0)	0 (0)	5 (33.3)	0 (0)	0 (0)
ST23 (9, 14.5)	1 (11.1)	0 (0)	1 (11.1)	0 (0)	0 (0)	0 (0)	2 (22.2)	0 (0)	0 (0)
Others STs (8, 13)	1 (12.5)	0 (0)	1 (12.5)	0 (0)	0 (0)	0 (0)	5 (62.5)	0 (0)	0 (0)

Oxa: oxacillin; Cip: ciprofloxacin; Ofl: ofloxacin; Lev: levofloxacin; Mox: moxifloxacin; Gat: gatifloxacin; Tob: tobramycin; Chl: chloramphenicol; Van: vancomycin.

### 3.7 Statistical differentiation between isolates from healthy conjunctiva, and ocular infection

**Fig 1:**
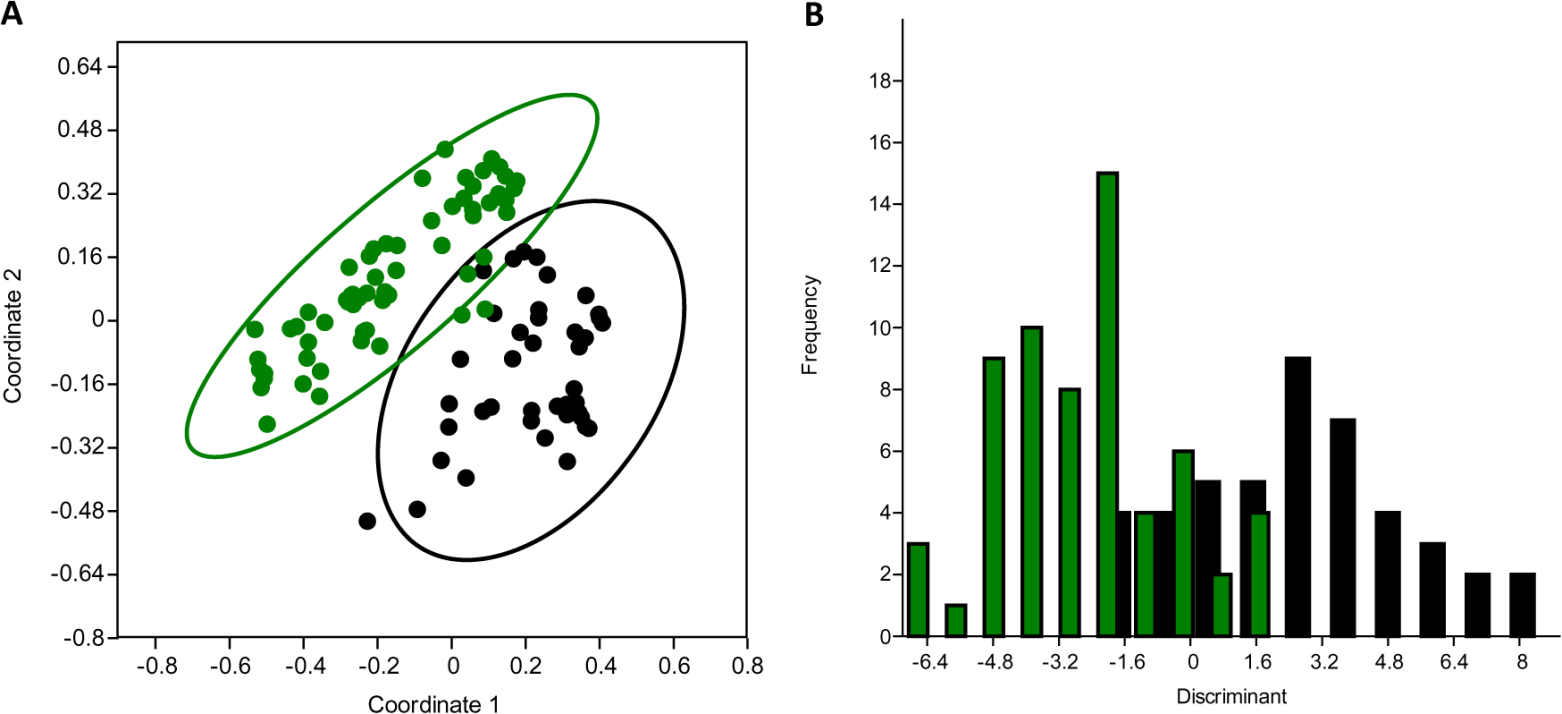
Statistical analysis of phenotypic and genotypic data. Principal Coordinates Analysis (PCoA) of *S*. *epidermidis* (A) from Ocular Infection (OI, green) and isolates from healthy conjunctiva (HC, black). The circles represent the confidence interval 95%. The first two coordinate axes represent 31% of the variance. Discriminant analysis (B) of the isolates of *S*. *epidermidis* from Ocular Infection (OI, green) and healthy conjunctiva (HC, black). Negative values belong to OI isolates and positive to HC isolates. The discriminant function properly allocated to 77% of isolates from HC and 84% from OI.

**Fig 2:**
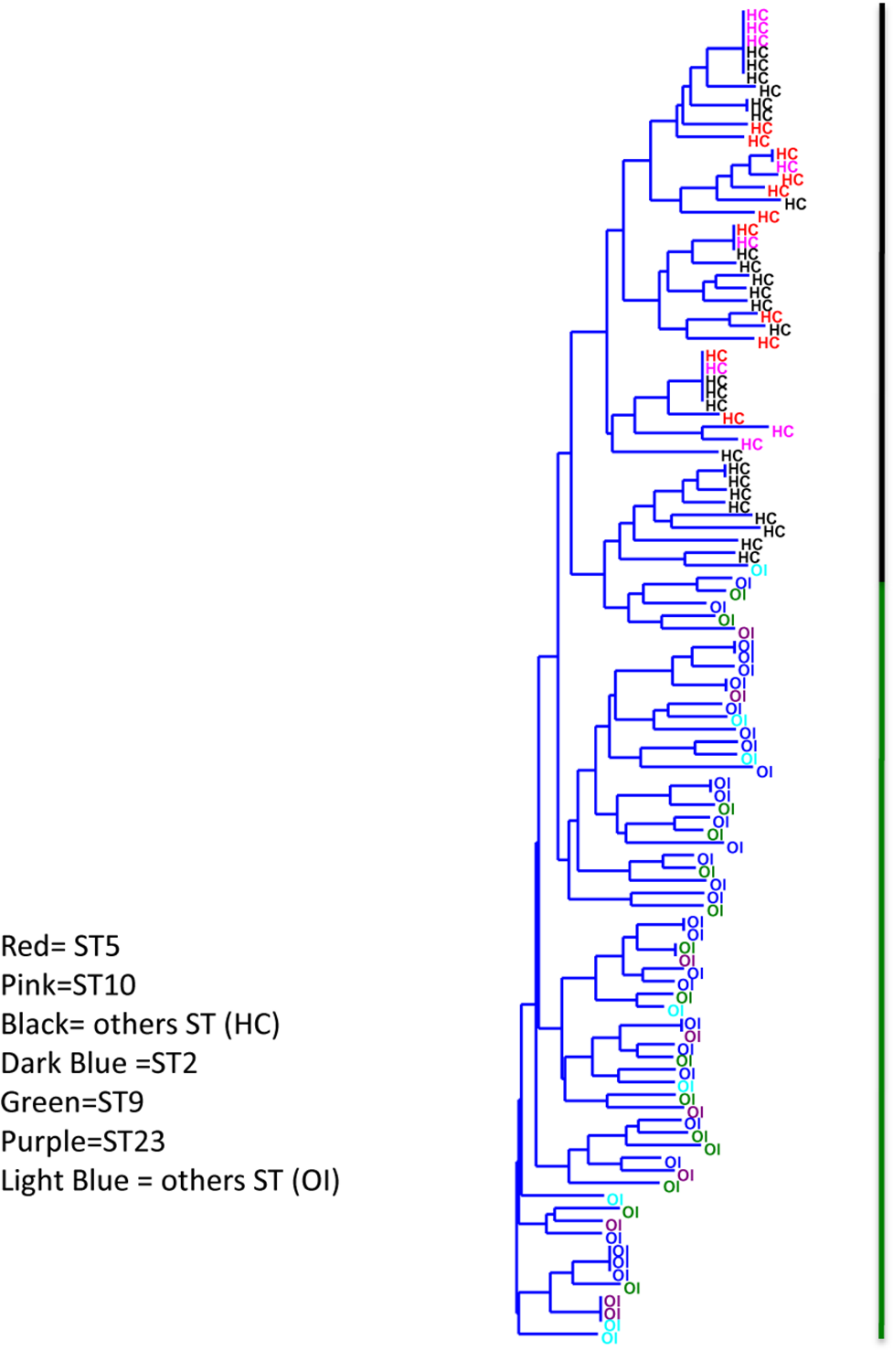
Cluster analyses of the isolates from Ocular Infection and healthy conjunctiva. The colors represent the ST's. The black vertical line represents the isolates from healthy conjunctiva (HC) and green vertical line isolates from ocular infection (OI).

It was observed that PCoA exhibited a clear separation between the two groups of isolates (using 2 axis had the highest percentage of representation, with 31% of explained variance) (Fig 1A). Similarly, with the discriminant analysis (DA), ocular infection isolates were grouped within the more negative values of the graph while healthy conjunctiva isolates among the most positive values (Fig 1B). With both methods, the isolates were separated in two groups. Subsequently, in order to find the preponderant biomarkers of the isolates, the coefficient of the discriminant function was determined, and a preponderant biomarker was considered when a value equal to 0.5 or greater than 0.5 was reached. Based on the above, ocular infection isolates are discriminating in the biomarkers of *agr* III (-5.5711) or *agr* II (-3.5108), SCC*mec* V (-3.3422) or SCC*mec* I (-3.2048), *mecA* gene (-2.2384), resistance to tobramycin (-1.7989), positive biofilm (-1.2738), and IS256^+^ (-0.94603). In contrast, healthy conjunctiva isolates were discriminating in *agr* I (3.8668), and resistance to chloramphenicol (3.7826), ciprofloxacin (2.5111), gatifloxacin (2.2844), oxacillin (1.49). Besides, it was found that the discriminant function properly assigned to 77% of healthy conjunctiva isolates and 84% of ocular infection isolates. Later, these data were present in a dendrogram to search a relationship with the lineage ST (Fig 2), two major groups were observed, the first includes all the isolates from healthy conjunctiva and their corresponding STs (ST5 and ST10 were predominant) and some isolates from ocular infection, and the second grouped all the ocular infection isolates with lineage ST2 and ST9.

## Discussion

*S*. *epidermidis* provides a helpful symbiotic relationship with the skin and conjunctiva of humans. Currently, *S*. *epidermidis* is recognized as an opportunistic pathogen in patients with low immune response and frequently cause infections linked to medical devises, particularly in cases of prosthetic cardiac valves, cerebrospinal fluid shunts, intravascular catheters and orthopedic implants [2]. To explain the ability of *S*. *epidermidis* as an opportunistic pathogen, some studies at genetic level had been performed. Genome sequencing of 17 isolates of *S*. *epidermidis* obtained from different parts of the body of a healthy individual showed significant differences [6], indicating the high genetic variation of this bacterium. Moreover, it have been documented the isolation of different strains of *S*. *epidermidis* from acquired infections at the same hospital. With regard to ocular infection isolates, the comparison of 42 isolates of keratitis and endophthalmitis with 14 healthy conjunctiva isolates, revealed 11profiles of fluorescence-amplified fragment lentgth polymorphism (FAFLP); a clear separation of groups was observed between ocular infection isolates (keratitis and endophthalmitis) and commensal isolates [18]. In this work, the PFGE generated a high discrimination level between isolates of the two sources of isolation and no accurate data that allow the separation between them were observed. Typing by MLST STs generated lineages that were shared between the two sources of isolation but some lineages were not shared. The lineage ST2 was the most frequent in ocular infection isolates (48.4%); however this lineage was not common in healthy conjunctiva isolates (6.6%), in which the ST5 lineage (24.4%) was the most abundant. This suggests that at STs level, there is a substantial difference between isolates from ocular infection and healthy conjunctiva, and that *S*. *epidermidis* that inhabits the conjunctiva probably could not infect the eye. Using a similar approach to this work in the hospital in Shangai, China compared clinical isolates from peripheral blood with nasal isolates from healthcare staffs and with community nasal isolates from healthy people, these authors found that the lineage ST2 was the most frequent in clinical isolates but this was absent in isolates from healthy patients [29]. The same result was reported in another hospital in Belgium [4]. Some studies have pointed out that the ST2 lineage of *S*. *epidermidis* is associated with virulent strains [30–32]. However, in a recent study, 36 isolates from keratitis (eye infection) generated lineages ST59, ST5 and ST6 mainly and these authors suggested that these lineages are occasional and could be specific for the eye infection [15]. The ST5 lineage has also been detected in clinical isolates of US hospitals [33] or in bone and joint infections [34]. These studies had demonstrated that there is an association between lineage ST5 and infections.

Biofilm production in *S*. *epidermidis* is a virulence factor and it is recognized that clinical isolates produce more biofilm than commensal isolates [4, 29]. Besides, the commensal isolates from conjunctiva produce more biofilm than the skin isolates [35]. Our results are consistent with these findings, because ocular infection isolates with lineage ST2 produced more biofilm than healthy conjunctiva isolates with ST5. The presence of the *icaA* and *icaD* genes (involved in the production of exopolysaccharide N-acetyl-glucosamine) and IS256 exhibited a low frequency in the isolates of the two sources of isolation; which it was according to *S*. *epidermidis* isolates from keratitis [15] and also *icaA* gene was not useful for discriminating between the isolates of keratitis from the healthy conjunctiva isolates [18]. However in *S*. *epidermidis* isolates from peripheral blood there was a correlation between the high production biofilm and the presence of *icaA* and *icaD* genes [30]. Furthermore quorum sensing activates the *agr* operon and it negatively regulates the biofilm production. This operon has three types of polymorphisms. In ocular infection isolates there was a major tendency toward *agr* III than in commensal isolates. Ocular infection isolates with lineage ST2 presented *agr* II and III. Li, *et al*. (2010) found that clinical isolates of *S*. *epidermidis* harbored *agr* I while commensal isolates were *agr* II [27]. Our results were in agreement with that study, in which the isolates with lineage ST2 of prosthetic joint infections (PJI) presented mainly *agr* I, but isolates with lineage ST215 were *agr* III [36]. In *S*. *epidermidis* isolates from peripheral blood with lineage ST2 were predominantly *agr* III [32]. Isolates with lineage ST215 were found in patients at hospitals in Sweden and Norway [37], indicating that ST215 could be associated with an increased pathogenicity and/or greater ability to survive in hospital environment [37]. Conversely, in this study ST215 is not present in any of the isolates.

Another relevant virulence factor of *S*. *epidermidis* is the resistance to antibiotics. Healthy conjunctiva isolates showed higher resistance to antibiotics than ocular infection isolates. Healthy conjunctiva isolates with ST5 lineage were more resistant to oxacillin than ocular infection isolates with ST2 lineage. Contrary to another studies where it was exhibited that *S*. *epidermidis* isolates from different clinical sources are more resistant to antibiotics than commensal isolates [32, 34]. However, it has been documented that near to 50% of healthy conjunctiva isolates of *S*. *epidermidis* have mutations in the *gyrA* and *parC* genes, which have been associated with resistance to fluoroquinolones [38]; contrary, ocular infection isolates harbored fewer mutations and reduced resistant to these antibiotics [39–40]. Moreover, healthy conjunctiva isolates significantly increased their resistance to azithromycin and fluoroquinolone after repeated exposure to them [41]. This suggests that the native healthy conjunctiva strains have been under increasing pressure by the use of antibiotics for prophylaxis or during the treatment of ocular infection caused by *S*. *epidermidis*, or by another bacteria.

Based on the statistical analysis (PCoA and DA) of genetic and phenotypic data, it was found that the discriminant biomarkers for ocular infection isolates were: *agr* III or *agr* II, SCC*mec* V or SCC*mec* I, *mecA* gene, resistance to tobramycin, positive biofilm and IS256^+^, and by MLST was the lineage ST2. In contrast, the discriminant biomarkers in healthy conjunctiva isolates were *agr* I, and resistance to chloramphenicol, ciprofloxacin, gatifloxacin, oxacillin, and by MLST was the lineage ST5. This result shows that ocular infection isolates are markedly different from healthy conjunctiva isolates, and suggests that *S*. *epidermidis* from healthy conjunctiva do not infect the eye. However, we do not reject that the isolates from healthy conjunctiva could infect the eye. This opinion is supported by the work of Bispo *et al*. (2014) who found that *S*. *epidermidis* isolates from keratitis gave ST59 or ST5 lineages, are high producers of biofilm, absence of *icaA* gene and IS256, *agr* I and a high resistance to antibiotics [15]. These biomarkers were very similar to our healthy conjunctiva isolates with lineage ST5. Furthermore, the authors of that work suggested that *S*. *epidermidis* that inhabits the healthy conjunctiva is responsible for infecting the eye, but this suggestion was based only on the hypothesis of the proximity of the conjunctiva to the eye, because they did not work with healthy conjunctiva isolates of *S*. *epidermidis*. Another study has shown that isolates of *S*. *epidermidis* that colonize the conjunctiva and the eye are responsible of the post-operative endophthalmitis [16]. In contrast, it has been shown a clear separation between the healthy conjunctiva isolates with the isolates from keratitis and endophthalmitis by RFLP analysis [18]. Another characteristic of *S*. *epidermidis* isolates that inhabit the healthy conjunctiva is that they are polyclonal and can change their genotypic traits in a short time (months) [19]. Probably, in the healthy conjunctiva there is a common ancestor of *S*. *epidermidis* (lineage ST5), which generates, by mutations, different genotypic variants (other lineages STs) in response to changes in the conjunctiva over time. Based on the above information and in order to explain the origin of *S*. *epidermidis* isolates that infect the eye, in this paper, discriminant biomarkers of ocular infection were examined in healthy conjunctiva isolates, and it was found that 3 healthy conjunctiva isolates [two with ST2 and another with ST9] (3/45, 6.66%) had similar genotypic and phenotypic characteristics to ocular infection isolates, therefore a small population from healthy conjunctiva could cause an ocular infection. Alternatively, isolates of *S*. *epidermidis* that inhabit the skin of body parts could be those infecting the eye because it has been documented that these are genotypically different [6].

## Conclusion

With these results we conclude that in the healthy conjunctiva (6.66%) inhabit a low population of *S*. *epidermidis*, which possess discriminant and similar biomarkers to that found in ocular infection isolates. This small population might potentially infect the eye. The major population (with different characteristics to ocular infection isolates) inhabiting conjunctiva probably do not infect the eye; in part by the innate immune system of this organ, which recognize, control and select these isolates for their survival.

## Supporting Information

S1 Table Additional ocular infection data. (PDF)

S2 Table Additional healthy conjunctiva data. (PDF)
