# Supplementary material for: Molecular and Phenotypic Characterization of Staphylococcus epidermidis Isolates from Healthy Conjunctiva and a Comparative Analysis with Isolates from Ocular Infection
Source: PLoS One. 2015 Aug 14;10(8):e0135964. doi: 10.1371/journal.pone.0135964 (PMC4537226; doi:10.1371/journal.pone.0135964)
Supplement: S1 Table — (PDF) [file pone.0135964.s001.pdf]

Ocular infection

Isolates	Infection type	ST	PFGE	BIOFILM	*icaA*	*icaD*	IS256	*agr*	Ox	Ci	Of	Le	Mo	Ga	To	Ch	Va	SCCmec	*mec A*
**35**	**C**	2	A	-	-	-	-	2	s	s	s	s	s	s	s	s	s	I, IV	+
**47**	**C**	2	B	+				2	s	s	s	s	s	s	s	s	s	II	+
**51**	**C**	2	C	-	-	-	-	2	s	s	s	s	s	s	s	s	s	II, IV	+
**53**	**C**	2	D	-	-	-	-	2	s	s	s	s	s	s	R	s	s	IV	+
**61**	**C**	2	E	+	+	+	+	3	s	s	R	s	s	s	R	s	s	II	+
**68**	**Others**	2	F	+	+	+	-	3	s	s	R	s	s	s	R	s	s	II	+
**71**	**C**	2	G	-	-	-	-	3	s	s	s	s	s	s	s	s	s	I	+
**93**	**E**	2	A	+	-	-	+	3	s	s	s	s	s	s	s	s	s	V	-
**106**	**C**	2	B	+	-	-	-	2	s	s	R	s	s	s	R	s	s	IV	-
**139**	**C**	2	H	-	-	+	+	1	s	s	s	s	s	s	s	s	s	I	+
**146**	**U**	2	I	+	-	-	-	2	R	s	R	s	s	s	R	s	s	I	+
**149**	**C**	2	J	+	-	-	-	2	R	s	s	s	s	s	R	s	s	I, IV	+
**160**	**Others**	2	D	-	-	-	-	3	s	s	s	s	s	s	s	s	s	I	-
**168**	**C**	2	K	-	-	-	-	2	s	s	s	s	s	s	s	s	s	II	+
**186**	**C**	2	L	-	-	-	-	3	s	s	s	s	s	s	s	s	s	I	+
**213**	**E**	2	B	-	+	+	-	2	s	s	R	s	s	s	s	s	s	III	+
**1660**	**C**	2	M	-	-	-	-	2	s	s	s	s	s	s	s	s	s	II	+
**1664**	**Others**	2	N	-	-	-	+	3	s	s	s	s	s	s	R	s	s	V	-
**1668**	**C**	2	E	-	+	+	-	3	s	s	s	s	s	s	s	s	s	V	+
**1682**	**C**	2	G	-	-	-	-	3	s	s	s	s	s	s	s	s	s	IV	+
**1689**	**C**	2	O	-	+	-	+	2	s	s	s	s	s	s	R	s	R	III	+
**1701**	**C**	2	P	-	-	-	+	3	s	s	s	s	s	s	s	s	s	V	-
**1774**	**Others**	2	Q	-	-	-	-	3	s	s	s	s	s	s	s	s	s	II	+
**1819**	**E**	2	I	-	-	-	-	3	s	s	s	s	s	s	s	s	s	II	-
**1980**	**E**	2	S	-	-	-	-	2	s	s	s	s	s	s	s	s	s	V	+

2009	E	2	R	-	-	-	+	3	s	s	s	s	s	s	R	s	s	V	-
2038	C	2	Q	+	-	-	-	1	R	s	s	s	s	s	R	s	s	III, IV	+
1782-2	C	2	A	-	+	+	-	2	s	s	s	s	s	s	s	s	s	I, IV	+
1784-2	C	2	C	+	-	-	+	3	s	s	s	s	s	s	s	s	s	III	+
1789-2	C	2	D	-	-	-	+	2	s	s	R	s	s	s	R	s	s	V	-
50	Others	9	A	-	-	-	-	1	s	s	s	s	s	s	s	s	s	II	+
56	E	9	C	+	-	-	-	3	s	s	s	s	s	s	s	s	s	II	-
58	C	9	E	-	+	-	-	2	s	s	s	s	s	s	s	s	s	IV	+
60	C	9	F	+	+	+	-	2	s	s	s	s	s	s	s	s	s	V	+
105	C	9	H	+	-	-	-	3	R	s	s	s	s	s	R	s	s	II	+
108	C	9	I	-	-	-	-	2	s	s	R	s	s	s	R	s	s	III, IV	+
144	U	9	K	+	-	-	+	3	s	s	s	s	s	s	s	s	s	V	-
155	C	9	M	+	-	-	-	3	s	s	s	s	s	s	s	s	s	V	-
214	C	9	O	+	-	-	-	2	R	s	s	s	s	s	s	s	s	V	+
215	C	9	E	-	+	-	+	3	s	R	R	s	s	s	R	s	s	II	+
1085	Others	9	P	-	+	-	-	3	s	s	s	s	s	s	s	s	s	V	+
1784	Others	9	S	-	-	-	+	3	R	s	s	s	s	s	s	s	s	II	+
2050	C	9	A	-	+	+	-	3	s	s	R	s	s	s	R	s	s	III	+
2938	Others	9	B	+	-	-	-	3	s	R	R	s	s	s	R	s	s	V	-
1864-2	U	9	S	-	+	+	-	3	s	s	s	s	s	s	s	s	s	V	-
54	E	23	A	-	-	-	-	3	s	s	s	s	s	s	s	s	s	V	+
95	E	23	C	+	+	+	-	3	s	s	R	s	s	s	R	s	s	II, IV	+
156	C	23	B	+	-	-	-	2	s	s	s	s	s	s	s	s	s	II, IV	+
159	Others	23	H	+	-	-	-	3	s	s	s	s	s	s	s	s	s	II	+
199	U	23	S	-	+	+	-	2	s	s	s	s	s	s	s	s	s	IV	+
983	Others	23	R	+	-	-	+	3	R	s	s	s	s	s	R	s	s	V	-
1700	C	23	Q	+	-	-	-	3	s	s	s	s	s	s	s	s	s	I	+
1843	Others	23	N	+	-	-	-	3	s	s	s	s	s	s	s	s	s	I	+

**2022**	**U**	23	M	-	-	-	-	3	s	s	s	s	s	s	s	s	s	V	-
**63**	**E**	16	L	+	-	-	-	2	s	s	R	s	s	s	R	s	s	III	+
**96**	**E**	10	K	+	-	-	+	3	R	s	s	s	s	s	R	s	s	I, IV	+
**90**	**Ohers**	87	F	-	-	+	-	3	s	s	s	s	s	s	R	s	s	I	+
**98**	**E**	38	E	-	-	-	-	2	s	s	s	s	s	s	R	s	s	I	+
**1654**	**U**	71	D	+	+	+	-	1	s	s	s	s	s	s	s	s	s	I	+
**1655**	**C**	21	B	+	-	-	-	3	s	s	s	s	s	s	R	s	s	II	+
**1864**	**C**	57	L	+	-	-	-	3	s	s	s	s	s	s	s	s	s	V	+
**1948**	**C**	46	M	+	-	+	+	3	s	s	s	s	s	s	s	s	s	III	+

C= conjunctivitis; E=endophthalmitis; U=ulcers.
Ox=oxacillin; Ci=ciprofloxacin; Of=ofloxacin; Le=levofloxacin; Mo=moxifloxacin; Ga=gatifloxacin; To=tobramycin; Ch=chloramphenicol; Vancomycin.
