# Supplementary material for: Molecular and Phenotypic Characterization of Staphylococcus epidermidis Isolates from Healthy Conjunctiva and a Comparative Analysis with Isolates from Ocular Infection
Source: PLoS One. 2015 Aug 14;10(8):e0135964. doi: 10.1371/journal.pone.0135964 (PMC4537226; doi:10.1371/journal.pone.0135964)
Supplement: S2 Table — (PDF) [file pone.0135964.s002.pdf]

Healthy conjunctiva

Isolates	ST	PFGE	BIOFILM	*icaA*	*icaD*	IS256	*agr*	Ox	Ci	Of	Le	Mo	Ga	To	Ch	Va	SCC*mec*	*mecA*
**7**	5	A	-	-	-	-	3	S	S	S	S	S	S	S	S	S	I	-
**8**	5	C	-	-	-	-	3	S	S	S	S	S	S	S	R	S	IV	+
**10**	5	D	-	-	-	-	2	S	R	I	S	S	S	S	R	S	I	+
**14**	5	A1	-	-	-	-	1	R	R	R	R	S	I	S	R	S	III	+
**15**	5	B1	-	-	+	-	1	S	S	S	S	S	S	S	S	S	III	+
**23**	5	C1	-	-	-	-	1	R	S	S	S	S	S	S	S	S	II	+
**25**	5	D1	-	-	-	-	1	R	R	R	R	S	S	S	S	S	I	-
**88**	5	T	-	-	+	+	1	R	S	S	S	S	S	S	R	S	III	+
**96**	5	A1	-	-	-	-	2	S	S	S	S	S	S	S	S	S	III, IV	-
**106**	5	C1	-	-	-	-	1	R	R	R	S	S	S	S	R	S	II	+
**129**	5	B1	-	-	-	-	2	R	R	R	S	S	S	I	S	S	V	+
**2**	10	F	-	-	-	-	3	S	S	S	S	S	S	S	S	S	V	
**4**	10	G	-	-	-	-	3	S	R	R	I	S	R	S	R	R	III	-
**9**	10	E1	-	-	-	+	2	s	s	s	s	s	s	s	s	s	II	+
**22**	10	F1	-	-	-	-	2	S	S	S	S	S	S	S	S	S	IV	-
**97**	10	G1	-	-	-	-	1	S	S	S	S	S	S	S	S	S	III	+
**100**	10	C1	-	-	-	-	1	S	S	S	S	S	S	S	S	S	II	+
**118**	10	E1	-	-	-	-	1	S	S	I	S	S	S	S	I	S	I	+
**130**	10	V	-	-	-	-	1	R	R	R	R	I	I	S	I	S	IV	-
**137**	10	F1	-	-	-	-	3	S	R	R	S	S	S	S	R	S	IV	+
**69**	238	I	-	-	-	+	1	S	S	S	S	S	S	S	S	S	I	+
**90**	238	J	-	-	-	+	1	S	S	S	S	S	S	S	S	S	II	+
**94**	238	H1	-	-	-	-	1	S	S	S	S	S	S	S	S	S	III	+
**146**	238	I1	-	-	-	-	2	S	S	S	S	S	S	S	S	S	V	+
**119**	118	J1	-	-	-	-	2	R	S	S	S	S	S	R	R	S	V	-
**126**	118	K1	-	-	-	-	2	R	I	I	S	S	S	S	S	S	IV	-

**128**	118	L1	-	-	-	-	2	**R**	**S**	**S**	**S**	**S**	**S**	**S**	**R**	**S**	**I**	+
**144**	118	M1	-	-	-	-	3	**S**	**S**	**S**	**S**	**S**	**S**	**S**	**S**	**S**	**II**	+
**120**	2	K	-	-	-	-	3	**S**	**S**	**S**	**S**	**S**	**S**	**S**	**S**	**S**	V	
**132**	2	A	+	+	+	-	2	**S**	**S**	**S**	**S**	**S**	**S**	**S**	**S**	**S**	**V**	+
**139**	2	A	+	-	+	-	2	**R**	**I**	**I**	**S**	**S**	**S**	**S**	**S**	**S**	**IV**	**-**
**11**	4	M	+	+	+	-	3	**R**	**S**	**S**	**S**	**S**	**R**	**S**	**R**	**I**	**II**	+
**92**	4	E1	-	-	-	-	1	**S**	**S**	**S**	**S**	**S**	**S**	**S**	**S**	**S**	**II**	+
**114**	4	H1	-	-	-	-	1	**S**	**S**	**S**	**S**	**S**	**S**	**S**	**S**	**S**	**I**	+
**30**	9	N1	-	-	-	-	1	**S**	**S**	**S**	**S**	**S**	**S**	**S**	**R**	**S**	**III**	+
**48**	9	O1	-	-	-	+	3	**R**	**S**	**S**	**S**	**I**	**S**	**R**	**R**	**S**	**II**	+
**152**	9	P1	+	+	+	-	2	**R**	**R**	**R**	**R**	**I**	**I**	**S**	**S**	**S**	**V**	**-**
**35**	23	N	-	-	+	+	1	**R**	**R**	**R**	**R**	**S**	**S**	**S**	**R**	**S**	**II**	**-**
**151**	23	J1	+	+	+	-	2	**R**	**S**	**S**	**S**	**S**	**S**	**S**	**S**	**S**	**IV**	**-**
**13**	26	Y	+	+	+	-	2	**R**	**S**	**S**	**R**	**R**	**R**	**R**	**S**	**S**	**II**	+
**17**	135	O1	-	-	+	-	2	**R**	**S**	**S**	**S**	**S**	**S**	**S**	**R**	**S**	**II, IV**	+
**29**	494	P	-	-	-	-	2	**S**	**R**	**R**	**I**	**S**	**S**	**S**	**R**	**S**	**II**	+
**31**	43	D1	+	+	+	-	1	**S**	**S**	**S**	**S**	**S**	**S**	**S**	**S**	**S**	**IV**	+
**103**	48	Z	+	+	+	-	2	**S**	**S**	**S**	**S**	**S**	**S**	**I**	**I**	**S**	**II**	+
**131**	173	L1	-	-	-	-	3	**S**	**S**	**S**	**S**	**S**	**S**	**I**	**S**	**S**	**IV**	**-**

Ox=oxacillin; Ci=ciprofloxacin; Of=ofloxacin; Le=levofloxacin; Mo=moxifloxacin; Ga=gatifloxacin; To=tobramycin; Ch=chloramphenicol; Vancomycin.
